# Long-term outcomes of modern radiation therapy for pituitary adenoma – different techniques: single institute experience

**DOI:** 10.1007/s11060-025-05228-1

**Published:** 2025-09-10

**Authors:** Alexandra Brand, Linda Agolli, Kerem Tuna Tas, Phillip Lishwiski, Markus Schymalla, Klemens Zink, Hilke Vorwerk, Ioanna Fragkandrea-Nixon, Thomas Held, Daniel Habermehl, Sebastian Adeberg, Ahmed Gawish

**Affiliations:** 1 Department of Radiotherapy and Radiation Oncology, Philipps- Universität Marburg, Marburg, Germany; 2 Department of Radiotherapy and Radiation Oncology, Marburg University Hospital, Marburg, Germany; 3 Marburg Ion-Beam Therapy Center (MIT), Department of Radiotherapy and Radiation Oncology, Marburg University Hospital, Marburg, Germany; 4 University Cancer Center (UCT) Frankfurt – Marburg, Marburg, Germany; 5 LOEWE Research Cluster for Advanced Medical Physics in Imaging and Therapy, (ADMIT), TH Mittel Hessen University of Applied Sciences, Giessen, Germany; 6 Department of Radiotherapy and Radiation Oncology, Giessen University Hospital, Giessen, Germany; 7 Department of Radiation Oncology, Heidelberg University Hospital, Heidelberg, Germany; 8 Heidelberg Institute of Radiation Oncology (HIRO), Heidelberg, Germany; 9 Beatson West of Scotland Cancer Center, Glasgow, UK

**Keywords:** Pituitary adenoma, Proton therapy, Radiotherapy

## Abstract

**Background:**

Pituitary adenomas are relatively common benign intracranial tumors that may cause significant hormonal imbalances and visual impairments. Radiotherapy (RT) remains an important treatment option, particularly for patients with residual tumor after surgery, recurrent disease, or ongoing hormonal hypersecretion. This study summarizes long-term clinical outcomes and radiation-associated toxicities in patients with pituitary adenomas treated with contemporary radiotherapy techniques at a single institution.

**Methods:**

A retrospective analysis was conducted on 122 patients treated with RT for pituitary adenomas at the tertiary Hospital in Germany between 1992 and 2023. Patient data were assessed for tumor characteristics, treatment modalities, and outcomes. Overall survival (OS), and local control (LC) were evaluated using Kaplan–Meier analysis. Statistical comparisons between subgroups were performed with the log-rank test. Treatment-related toxicities were graded according to the Common Terminology Criteria for Adverse Events (CTCAE), version 5.0.

**Results:**

Median follow-up was 107 months from initial diagnosis and 63 months post-RT. Most patients (96%) received fractionated stereotactic radiotherapy (FSRT), and 4% underwent single-session radiosurgery. RT achieved LC rates of 95% and 75% at 5 and 20 years, respectively. Proton therapy significantly improved LC and overall survival (OS) compared to photon-based treatments (*p* < 0.01). Hypopituitarism was the most common long-term toxicity, occurring in 40% of patients, while visual impairments were rare (< 3%). Tumor recurrence occurred in 9% of patients, primarily in those treated with delayed RT after incomplete resection.

**Conclusion:**

Modern RT techniques, particularly proton therapy, provide durable tumor control and manageable toxicity profiles for pituitary adenomas. Optimized timing and precision in RT delivery are critical to enhancing outcomes and minimizing complications. Long-term follow-up remains essential to monitor disease progression and late toxicities.

## Introduction

Pituitary adenomas represent a significant proportion of primary intracranial tumors, accounting for roughly 10–15% of all cases [1, 2]. These tumors may be classified according to their hormonal activity as either functioning (hormone-secreting) or non-functioning adenomas [2]. Clinically, they often contribute to pituitary dysfunction, presenting either through hormone excess syndromes or through deficiencies resulting from compression of normal pituitary tissue [3]. Visual symptoms are also common, particularly in macroadenomas, due to involvement of the optic apparatus or extension into the cavernous sinus, which may affect ocular motor function [4].

Radiotherapy (RT) plays a well-established role in the management of pituitary adenomas, especially in cases where surgery is incomplete, not feasible, or when hormonal normalization is not achieved postoperatively [5, 6]. The optimal timing of RT, particularly the value of early adjuvant therapy following surgery, remains a subject of ongoing clinical discussion [5]. While effective in tumor control, RT targeting the sellar region is frequently associated with long-term endocrine complications, most notably hypopituitarism [7–9]. The onset of radiation-induced hormonal deficits varies widely, from as early as one year to more than a decade post-treatment [10, 11]. Furthermore, specific radiation dose thresholds for different hormone-secreting cell types have been established, with growth hormone deficiencies often appearing first [9]. Other potential adverse effects of RT, such as visual dysfunction [12], cerebral radiation injury [13], and secondary tumor formation [1, 14], have led to ongoing discussions about its use in treating benign pituitary tumors Several clinically relevant aspects of radiotherapy (RT) for pituitary adenomas remain a matter of ongoing investigation. These include determining the most appropriate timepoint for intervention—whether as primary treatment, postoperative adjuvant therapy, or salvage after recurrence—as well as selecting patient subgroups who may benefit most, such as those with secreting versus non-secreting tumors. Furthermore, the comparative value of available RT modalities, including fractionated stereotactic techniques, radiosurgery, and intensity-modulated approaches, continues to be explored [5, 15–17]. Although numerous studies have described the short- and intermediate-term effects of RT in this context [18–20], long-term data—particularly with respect to tumor control and late toxicities—remain limited. Earlier reports have primarily focused on near-term outcomes [2, 21–23], leaving a gap in understanding the durability and safety of treatment over extended follow-up.

This study aims to address that gap by reporting our institution’s long-term experience with RT in a cohort of 122 patients with pituitary adenomas, offering insight into clinical outcomes and radiation-associated adverse effects over a period of several decades.

## Patients and methods

### Study design

After the acceptance of the ethics committee, we conducted a retrospective analysis of 122 patients diagnosed with pituitary adenomas who had undergone radiotherapy in our department at the university medical center in Germany. All the data were collected from the patient’s charts. Every patient included in this analysis was older than 16 years when receiving radiotherapy (RT) as a treatment for their diagnosed pituitary adenoma between 1992 and 2023. Pregnancy was an exclusion criterion. The median follow-up duration from the time of diagnosis was 107 months (range, 9–464 months). From the initiation of radiotherapy, the median follow-up was 63 months (range, 4–243 months).

### Patient characteristics

At the time of first diagnosis, 11 patients (9% out of 122) had no reported symptoms while 111 patients (91% out of 122) presented with at least one clinical manifestation. Out of the symptomatic patients, 54 patients (44%) presented visual impairments, 18 patients (15%) suffered from headaches, 2 patients (2%) experienced nausea, and 48 patients (39%) showed endocrine symptoms. Among the patients with endocrine symptoms, 24 (20%) exhibited signs of elevated hormone excess, and 24 (20%) had a hypofunction of the pituitary gland. Patient characteristics are summarised in Table 1.

**Table 1:** Summary of patient characteristics

Patient characteristics		
Biometric	(years)	(range)
Median age	56	16–79
	(n)	(%)
Female	73	60
Male	49	40
Initial symptoms	(n)	(%)
Asymptomatic	11	9
Pituitary subfunction	25	20
Hormonal excess	24	20
Reduced visual field/acuity	54	44
Chiasma opticum compression	55	45
Cerebral symptoms: headache	18	15
Cerebral symptoms: nausea	2	2
Histology	(n)	(%)
Non-secreting	80	66
GH-secreting	20	16
Prolactin-secreting	6	5
ACTH-secreting	4	3
TSH-secreting	3	2
Others	9	7
Surgery	(n)	(%)
Complete	56	46
Incomplete	61	50
No surgery	5	4
Re-surgery	(n)	(%)
Complete	25	20
Incomplete	24	20
No re-surgery	73	60
Radiotherapy	(n)	(%)
SRS	5	4
FSRT	111	91
	(Gy)	(range)
Median single dose	1.8	1.8–16
Median total dose	50.4	50-59.4
	(n)	(%)
Primary definitive RT	5	4
Post-operative RT	28	23
RT for recurrent tumors	89	73

### Histopathological findings

Non-secreting pituitary adenomas were found in 80 patients (66%). Through histological and blood hormone analysis, secreting adenomas were identified in 42 patients (34%), including 20 GH-secreting (48%), 6 prolactin-secreting (14%), 4 ACTH-secreting (10%) and 3 FSH-secreting adenomas (7%).

### Surgery

In the present analysis, 5 patients (4%) were subjected to RT as primary treatment due to an inoperable pituitary adenoma. Also, 117 patients (96%) had at least one surgery before their RT. Due to recurrent adenomas, 39 patients had one re-surgery after the initial surgery before receiving RT (33% out of 117). More than two surgeries before RT were performed on 9 patients (8% out of 117). Out of all the patients who underwent surgery, 25 patients have been treated with RT within 6 months as an adjuvant treatment line (21% out of 117) and 89 patients after more than 6 months (76% out of 117) after their last surgery. In 51 cases, a complete resection of the adenoma before the RT was performed (44% out of 117), while for 66 patients, surgery was deemed incomplete (56% out of 117).

### Radiotherapy

Treatment planning was based on computed tomography (CT) imaging in all patients, with MRI co-registered when available to improve target delineation and patient positioning. Immobilization was performed using individualized thermoplastic masks to minimize movement during therapy. Radiation delivery was planned using three-dimensional conformal techniques with raster-scanning methodology. Depending on tumor location and proximity to critical structures, either Single-Beam Optimization (SBO) or Intensity-Modulated Proton Therapy (IMPT) was selected to optimize dose conformity and reduce exposure to surrounding healthy tissue. The majority of patients (*n* = 117; 96%) were treated with fractionated radiotherapy, while a small subset (*n* = 5; 4%) received stereotactic radiosurgery (SRS). Given the limited number, no separate outcome analysis was performed for this subgroup, and these patients were included descriptively in the overall cohort to provide a complete overview of radiotherapy practices at our institution. The median total radiation dose was 50.4 Gy (range: 50–59.4 Gy), with a median single dose of 1.8 Gy per fraction (range: 1.8–16 Gy) and a median number of 28 fractions (range: 1–33). In summary, the majority of patients were treated with a fractionated regimen of 50.4 Gy in 28 fractions (1.8 Gy per fraction). Dose and fractionation were adapted in selected cases according to tumor size, location, and proximity to critical structures, with all treatments planned and delivered according to institutional protocols.

### Follow-up

Following radiotherapy, patients underwent structured follow-up beginning approximately 12 weeks after treatment. Each visit included neurological assessment and contrast-enhanced brain MRI, performed every 3–6 months during the early post-treatment years and subsequently at up to 12-month intervals. Patients were seen either at our department in Marburg or by their treating physician in private practice. Follow-up evaluations included tumor assessment regarding regression or progression, endocrinological status measured by blood chemistry, and documentation of visual deficits. Progression in the field was defined as enlargement of the adenoma, which was detected through imaging performances such as MRI or CT and a new increasing hormone level.

### Statistical analysis

Local control (LC) was defined as the time interval between the first radiotherapy session and the date on which local tumor progression within the treated field was confirmed. To enhance the precision of the overall survival (OS) evaluation, the following parameters were established: OS since primary diagnosis was calculated from the date of the initial adenoma diagnosis to the date of the most recent follow-up or the date of death expressed in months. OS since the radiotherapy was determined from the date of the first radiotherapy session to the date of last follow-up or death, also expressed in months. Survival analyses were performed using the Kaplan–Meier method. For statistical comparison of survival curves between patient subgroups, the log-rank test was applied. Multivariate analyses such as Cox regression were not conducted due to the retrospective design. All statistical evaluations were carried out using the software tool SPSS 29.1. A p-value of less than 0.05 (*p* < 0.05) was established as the threshold for statistical significance.

### Toxicity assessment

Radiotherapy toxicity refers to the adverse effects on healthy tissues caused by radiation exposure during cancer treatment, possibly resulting in both early (< 6 months post-RT) and late toxicities (> 6 months post-RT). Early and late toxicities were prospectively documented and graded according to the Common Terminology Criteria for Adverse Events (CTCAE), version 5.0. Acute toxicities included nausea, vomiting, dizziness, headache, hiccups, cognitive disturbance, alopecia, fatigue, and radiation dermatitis. Late toxicities included alopecia, central nervous system necrosis, vertigo, cognitive disturbance, seizures, headache, and vision loss.

## Results

### Toxicity

Within the first six months following radiotherapy, only 8 out of 122 patients (7%) reported experiencing grade 1–2 nausea and 3 patients (2%) indicated grade 1–2 vomiting. While no patient developed hiccups, 5 patients (4%) experienced episodes of vertigo, and only 2 of the 122 patients (2%) showed grade 1 cognitive disturbance according to CTCAE v5.0. Furthermore, 17% of the patients (21 out of 122) suffered from grade 1–2 headaches and an equivalent number indicated feelings of fatigue. Grade 1–2 erythema was observed in 11 patients (9%) while alopecia was documented in 27 out of 122 of the patients (22%). In the context of late toxicity evaluation, more than 6 months after radiotherapy, no case of hair loss was observed, and central nervous system necrosis only occurred in one patient (1%). Besides, 2 patients experienced grade 1 vertigo (2%), and 9 out of 122 patients (7%) suffered from grade 1–2 headache. Moreover, grade 1–2 cognitive disturbance were indicated by 5 out of 122 of the patients (4%).

### Survival and prognostic factors

**Fig. 1:**
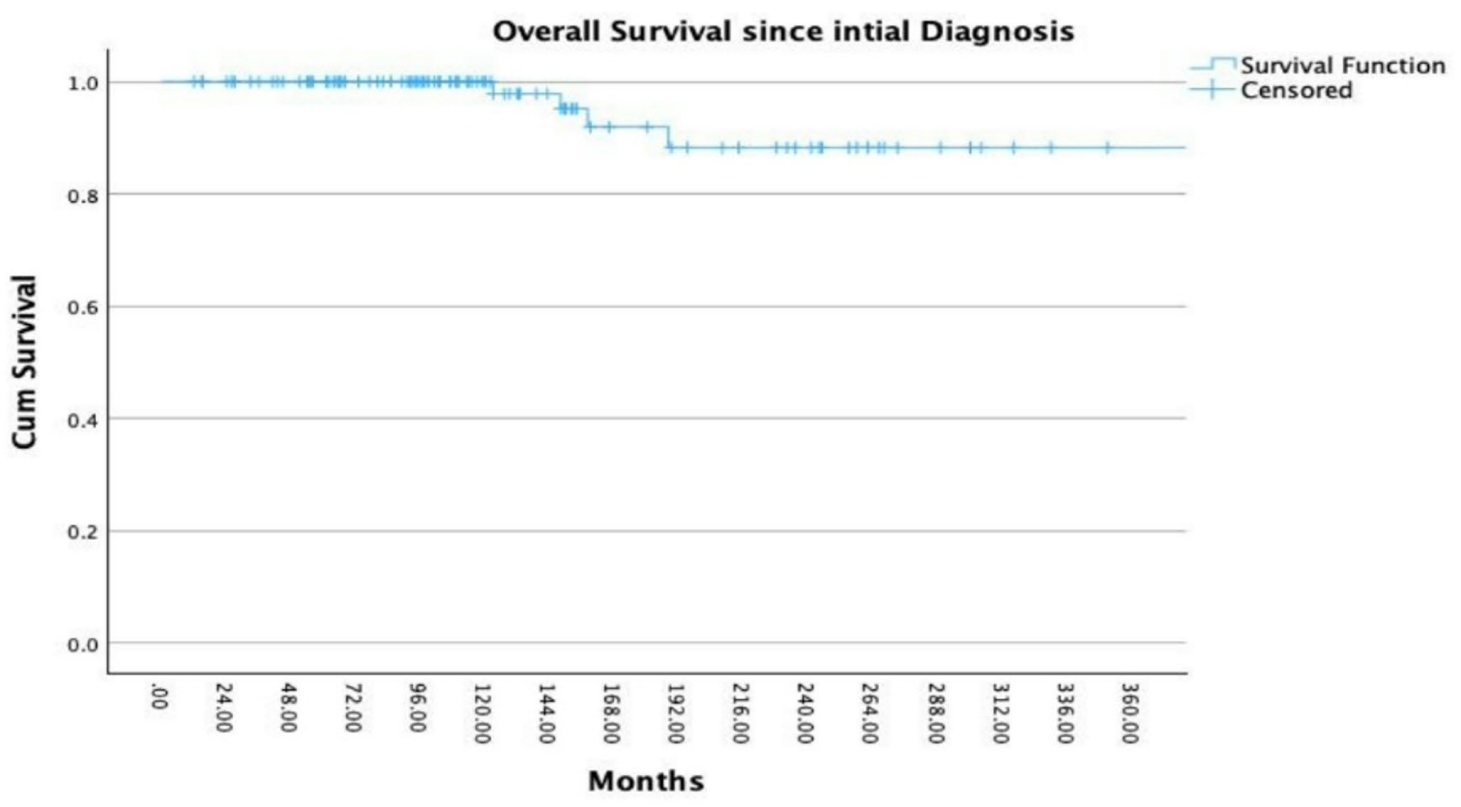
Overall survival (OS) in 122 patients with pituitary adenoma after initial diagnosis: OS rates were 100%, 97%, 88%, and 88% at 12, 120, 180, and 240 months. The x-axis shows time since diagnosis (months), and the y-axis shows cumulative overall survival probability. Censored observations are indicated by tick marks

**Fig. 2:**
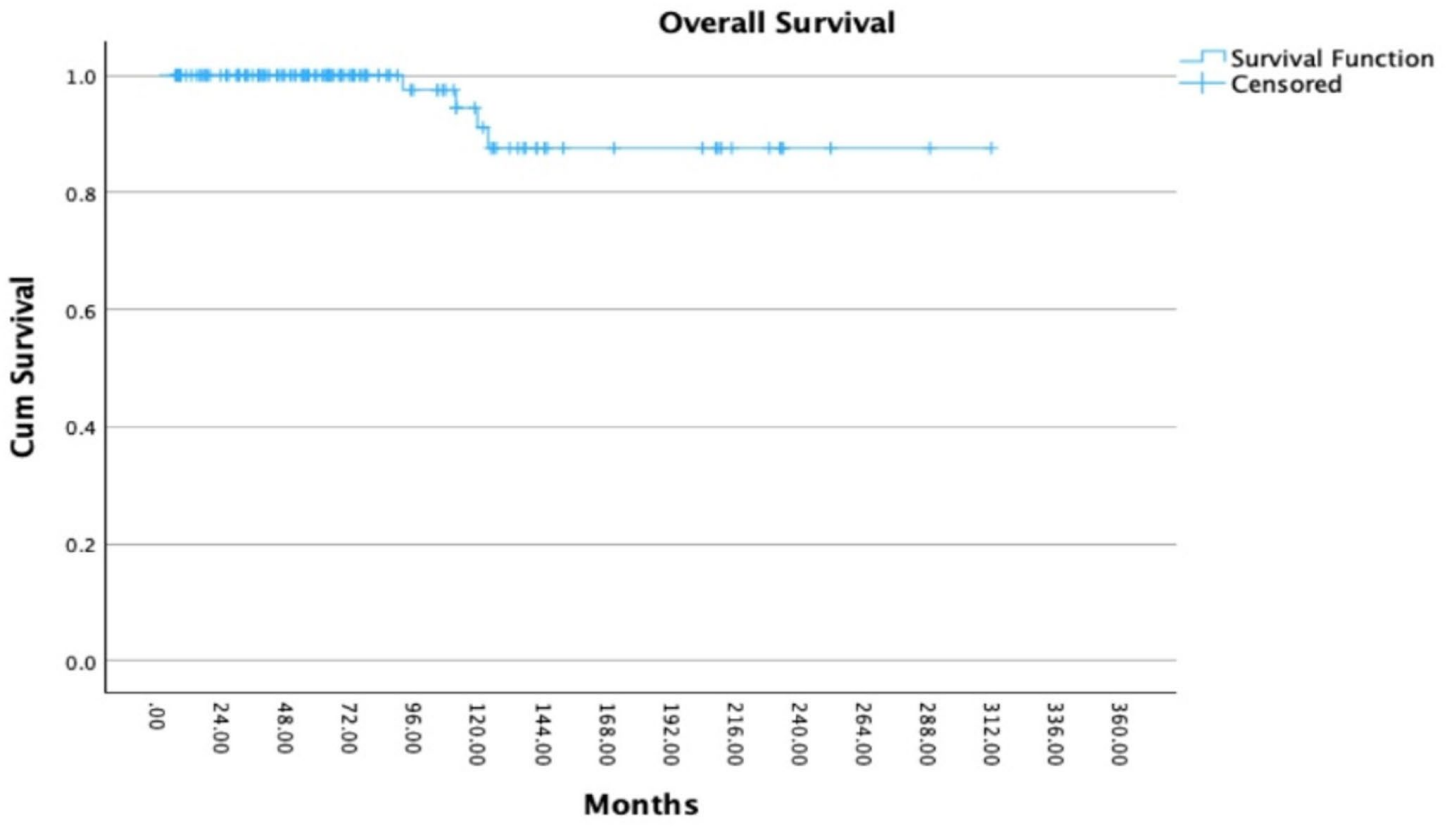
Overall survival (OS) in 122 patients with pituitary adenoma after radiotherapy (RT): OS rates were 100%, 87%, 87%, and 87% at 12, 120, 180, and 240 months. The x-axis indicates time since radiotherapy (months), and the y-axis shows cumulative overall survival probability. Censored cases are marked with tick symbols

The median follow up period from the time of diagnosis was 107 months (range 9–464 months) and the median follow-up time since RT amounted to 63 months (range 4–243 months). In addition, the median time from initial diagnosis to RT was 30.5 months (range 0–345). OS since the first diagnosis was 100%, 97%, 88%, and 88% at 12, 120, 180, and 240 months (Fig. 1). OS after RT was 100%, 87%, 87%, and 87% at 12, 120, 180, and 240 months (Fig. 2).

**Fig. 3:**
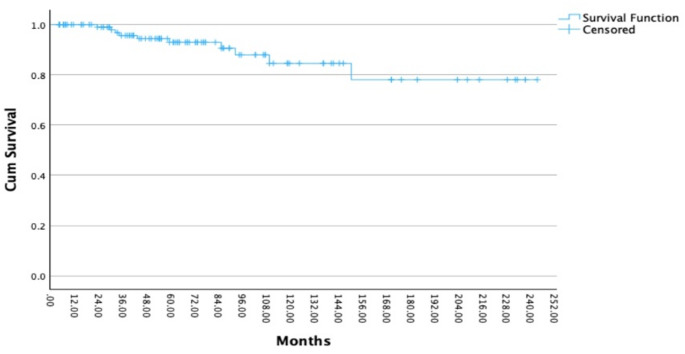
Local control (LC) in 117 patients with pituitary adenoma after initial surgery: LC rates were 100%, 84%, 78%, and 78% at 12, 120, 180, and 240 months. Complete resection before RT was associated with improved LC (*p* = 0.07). The x-axis indicates time since surgery (months), and the y-axis shows cumulative overall survival probability. Censored observations are indicated by tick marks

**Fig. 4:**
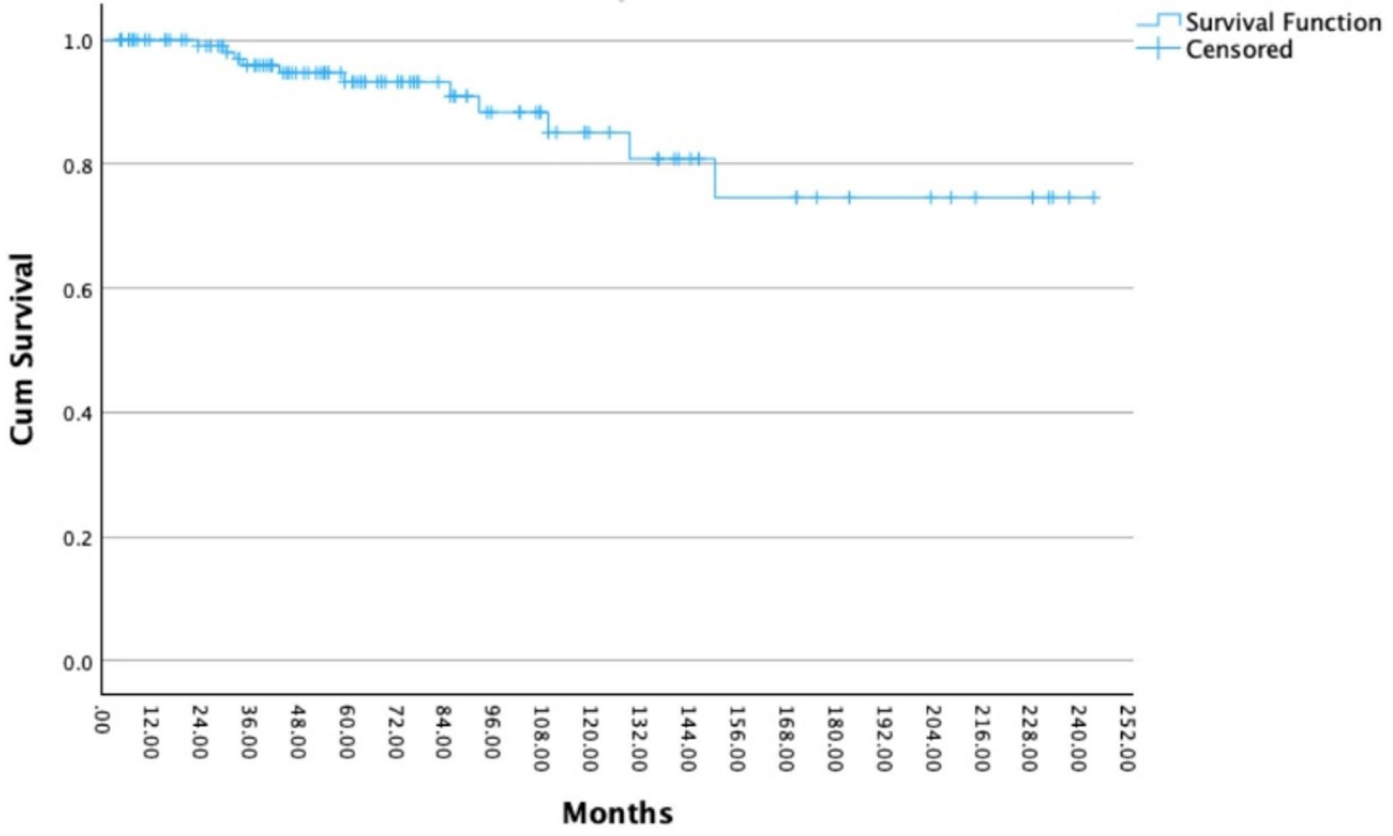
Local control (LC) in 122 patients with pituitary adenoma after radiotherapy (RT): LC was 100%, 95%, 86%, 75%, and 75% at 12, 60, 120, 180, and 240 months. The x-axis indicates time since radiotherapy (months), and the y-axis shows cumulative overall survival probability. Censored observations are indicated by tick marks

The statistical analysis showed that RT with protons as a treatment for pituitary adenomas has a significant influence on OS since RT (*p* < 0.001). Moreover, there is a significant correlation between RT with protons and LC (*p* < 0.001). The different characteristics of the initial adenoma about the secretion of hormones or the non-secretion of hormones had no significant effect on the LC (*p* = 0.095). Of 122 patients, 117 (96%) underwent at least one initial surgery before RT. LC after initial surgery was 100%, 84%, 78%, and 78% at 12, 120, 180, and 240 months (Fig. 3). In 51 out of 117 patients (44%), a complete resection of the pituitary adenoma before RT was possible, and 66 out of 117 patients (56%) received an incomplete resection. This analysis proves a significant correlation both between a complete resection and LC (*p* = 0.007) (Fig. 3) and between a complete resection and OS since initial diagnosis (*p* = 0.027). LC after radiotherapy was 100%, 95%, 86%, 75%, and 75% at 12, 60, 120, 180, and 240 months (Fig. 4).

### Patterns of relapse

Tumor-relapse was observed in 11 out of 122 patients (9%), generally within a median time of 59 months post-RT (range 34–150 months). Each tumor recurrence was identified through radiological follow-ups using MRI or CT scans as the primary diagnostic tools. No endocrinological relapse was observed in any of the 122 patients following radiotherapy. In 7 out of the 11 cases of recurrent adenomas (64%), the initial tumor was found to be non-secreting at the time of first diagnosis, whilst in 4 out of the 11 recurrent adenomas (36%), the initial tumor was secreting. The statistical analysis has shown a significant correlation between secreting adenomas and progression (*p* = 0.013). With regard to the different treatment groups, the following was noted: 8 out of 11 patients (73%) with tumor recurrence received late postoperative RT, which was defined as latency of more than 6 months between surgery and RT. This indicates that the majority of patients with relapse received late post-operative RT, as in most cases the initial strategy after surgery was active surveillance and radiotherapy was administered only at the time of documented tumor progression without additional surgery. Also, 2 patients (18%) who suffered from adenoma recurrence underwent early postoperative RT which was characterized by a time period of less than six months between surgery and the start of radiation therapy. Only one out of 11 patients (9%) with tumor recurrence received RT solely without prior surgery. In general, tumor relapse is mainly detected in patients who received RT at the age of 40 or older (Table 2).

**Table 2:** Effect of age on tumor control

	No. of patients	No. of tumor relapse (%)
Age (years)		
16–19	3	0 (0%)
20–29	4	0 (0%)
30–39	14	0 (0%)
40–49	24	3 (13%)
50–59	28	5 (18%)
60–69	37	3 (8%)
70–78	12	0 (0%)

## Discussion

This study reinforces the pivotal role of radiotherapy (RT) in managing pituitary adenomas, demonstrating excellent long-term local control (LC) rates and manageable toxicity profiles. The observed 20-year local control rate of 75% is in line with outcomes reported in comparable long-term studies and reflects the effectiveness of current radiotherapy approaches in managing pituitary adenomas. Notably, patients in our cohort who received postoperative RT shortly after surgery — particularly those with residual tumor — showed lower rates of recurrence. Although this observation is based on a small subgroup and may be influenced by additional factors, it underscores the potential clinical relevance of timely RT administration. When applied early and with advanced techniques such as proton therapy, radiotherapy may offer enhanced disease control while maintaining a favorable toxicity profile, although further studies are warranted to confirm this observation. Our findings must be interpreted in light of the retrospective design. As a result, treatment protocols could not be reconstructed in detail for each patient (Table 3).

**Table 3:** Comparison of the recent studies for radiotherapy of pituitary adenoma

Study	Patient Number	Follow-up	Local Control (LC)	Tumor Recurrence	Dose (Gy)	Hypopituitarism (%)	Visual Impairment (%)
Our Manuscript	122	111.5 months	95% (5y), 75% (20y)	9%	50.4	40	< 3
Rieken et al. (2013)	132	10 years	90.4% (10y), 75.5% (20y)	Not reported	45–54	10.9	5.4
Scheick et al. (2016)	75	10 years	96% (non-secretory)	Not reported	45–50	26	None
Sathe et al. (2023)	75	5.25 years	91.2% (5y)	Not reported	45–50	20.6	4
Wattson et al. (2014)	165	43 months	98%	Not reported	45–54	45 (3y), 62 (5y)	Rare
Minniti et al. (2021)	120	10 years	90% (10y)	10%	45–54	30	< 5
Castinetti et al. (2020)	100	10 years	90% (10y)	10%	45–50	30	< 5

**Fig. 5:**
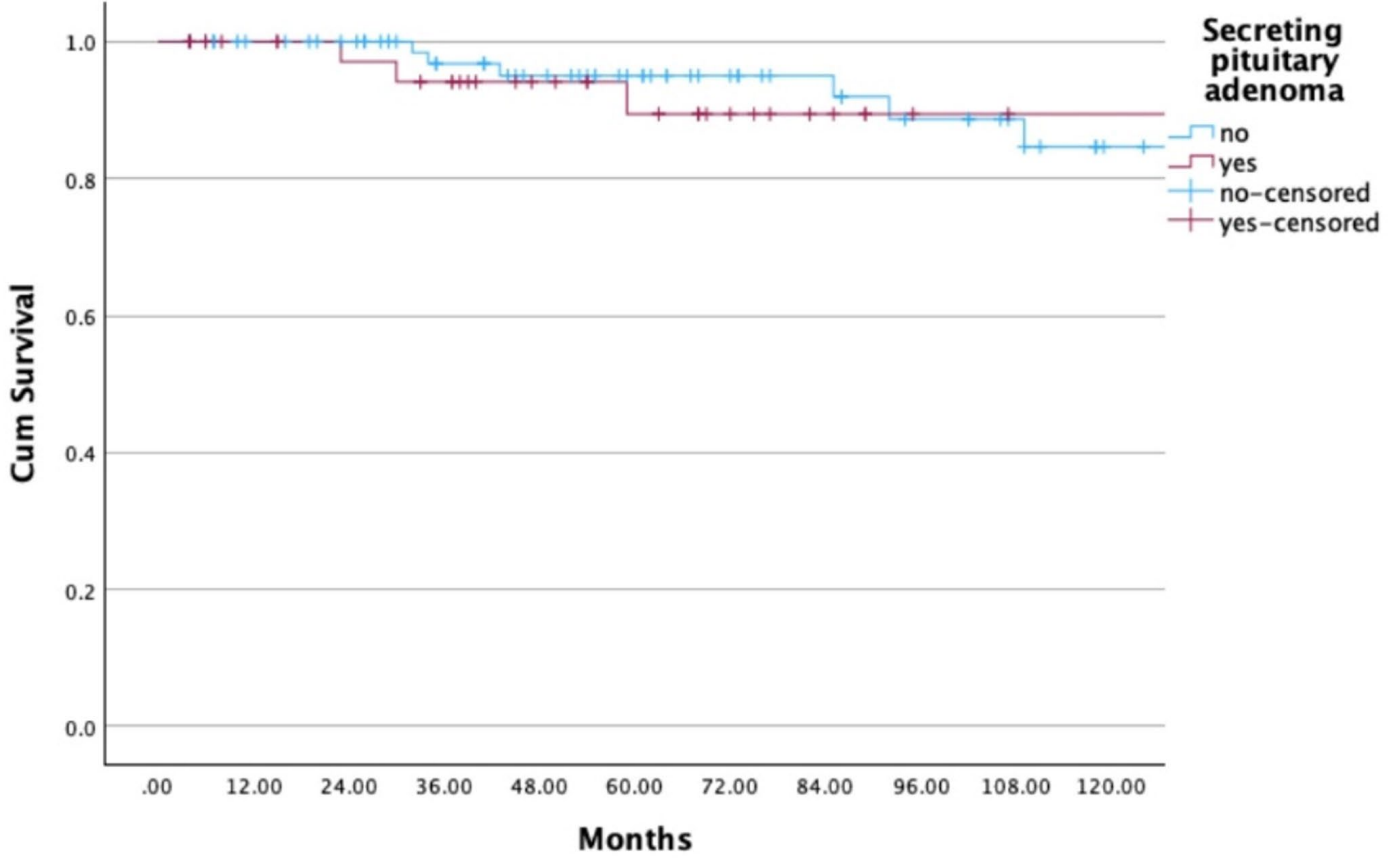
Local control (LC) in 122 patients with pituitary adenoma (secreting vs. non-secreting). Patients with secreting adenomas (pink line) showed a significantly reduced local control compared to those with non-secreting tumors (blue line) (*p* = 0.013). Of the 11 patients who experienced tumor recurrence, 7 (64%) had non-secreting adenomas and 4 (36%) had secreting adenomas at initial diagnosis. The x-axis indicates time since radiotherapy (months), and the y-axis shows cumulative local control probability. Censored observations are indicated by tick marks

**Fig. 6:**
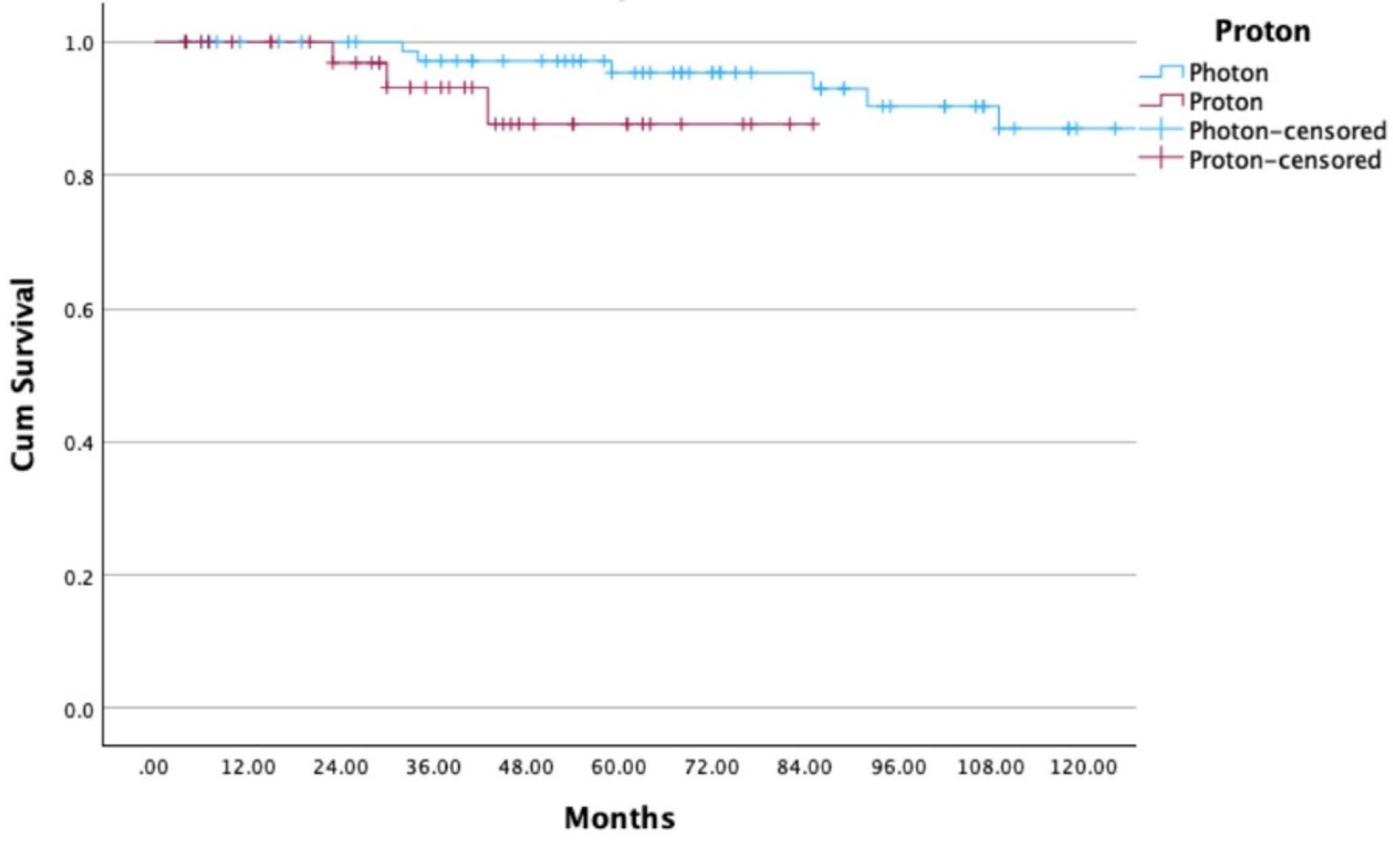
Local control (LC) in 122 patients with pituitary adenoma (photon and proton radiotherapy). The x-axis indicates time since radiotherapy (months), and the y-axis shows cumulative local control probability. Censored observations are indicated by tick marks

The study by Rieken et al. [24] analyzed long-term outcomes of RT for pituitary adenomas, reporting local progression-free survival (LPFS) rates of 90.4% at 10 years and 75.5% at 20 years, with overall survival (OS) rates of 93.3% and 61.0% at these intervals, respectively. These findings are consistent with our results, which showed LC rates of 95% at 5 years and 75% at 20 years. Both studies emphasize the benefit of early postoperative RT, demonstrating better tumor control than delayed intervention. However, hypopituitarism was less frequent in Rieken’s cohort (10.9% post-RT) compared to our cohort (40%), possibly reflecting differences in patient selection, follow-up duration, or radiation modalities. Visual impairments were also more common in their cohort (5.4%) than in ours (< 3%), highlighting the advantages of modern precision techniques, including proton therapy, which demonstrated superior LC and OS in our study. Both studies reported that radiotherapy had no relevant negative impact on quality of life (QoL), with most patients maintaining stable or improved scores throughout follow-up. These results reflect advances in radiotherapy, particularly proton therapy, which aim to enhance local tumor control while minimizing toxicity (Figs. 5 and 6).

In a study by Scheick et al. [25], long-term outcomes after fractionated RT for pituitary adenomas were stratified by secretory status. Patients with non-functioning tumors achieved a 10-year clinical control rate of 96%, whereas secretory adenomas were associated with significantly lower biochemical and radiological control rates (62%), primarily due to persistent hormonal hypersecretion. This disparity highlights the need for higher RT doses or alternative techniques for secretory tumors. Hypopituitarism developed in 26% of patients, primarily affecting cortisol and thyroid hormone secretion. Notably, there were no radiation-induced secondary malignancies or visual complications, emphasizing the safety of moderate-dose fractionated RT (45–50 Gy). Our study similarly reported excellent long-term local control for pituitary adenomas but identified proton therapy as offering superior outcomes compared to photon-based techniques. Our findings of hypopituitarism in 40% of the patients were higher than in the Scheick study, possibly due to differences in dosimetry, patient selection, or follow-up protocols. Additionally, our cohort exhibited lower rates of visual toxicity (< 3%), reflecting the advantages of modern RT modalities, particularly proton therapy. These comparisons underscore the benefits of advanced techniques, like proton therapy, in improving outcomes, particularly for challenging cases such as secretory tumors.

The study by Sathe et al. [26] compared early versus delayed fractionated stereotactic radiotherapy (FSRT) for nonfunctioning pituitary adenomas (NFPA) in 75 patients. Tumor control rates were excellent for both approaches, with a 5-year progression-free survival (PFS) of 91.2%. New hypopituitarism occurred in 20.6% of patients overall, with slightly higher rates in the early adjuvant group (22.2%) compared to the delayed group (18.8%). Notably, delaying FSRT extended the time to the onset of endocrinopathies without affecting tumor control or visual outcomes. The study concluded that while early FSRT is effective, reserving it for progressive disease may delay complications in selected cases, supporting a tailored approach based on individual tumor progression and clinical factors. Our findings align with these results, as we also observed that early postoperative RT was associated with better tumor control, particularly in cases of incomplete resection. However, our higher rate of hypopituitarism (40%) may reflect a longer follow-up period or more comprehensive endocrine monitoring, suggesting that late-onset hormonal deficiencies may be more common than previously reported.

Recent studies have further highlighted the evolving role of advanced RT techniques in managing pituitary adenomas. Minniti et al. [27] reviewed modern RT approaches for pituitary adenomas, including proton therapy and stereotactic radiosurgery (SRS). They reported that proton therapy, in particular, offers superior dose distribution, reducing the risk of toxicity to surrounding critical structures such as the optic chiasm and hypothalamus. Their findings support our observations that proton therapy is associated with lower rates of visual impairment and hypopituitarism compared with conventional photon-based RT. Additionally, they emphasized the importance of individualized treatment planning, particularly for secretory tumors, which often require higher radiation doses to achieve biochemical control.

A study by Castinetti and colleagues [28] investigated long-term outcomes following fractionated stereotactic radiotherapy (FSRT) in patients with pituitary adenomas, with particular attention given to endocrine control and patient-reported quality of life measures. They reported that FSRT achieved excellent tumor control rates, with 90% of patients remaining progression-free at 10 years. However, they also noted that hormonal deficiencies, particularly hypopituitarism, developed in 30% of patients, which is consistent with our findings. The study highlighted the importance of regular endocrine follow-up to monitor and manage late-onset hormonal deficiencies, which can significantly impact patient quality of life. These findings align with our emphasis on the need for long-term follow-up and comprehensive endocrine monitoring in patients undergoing RT for pituitary adenomas.

Hypopituitarism remains the most common long-term toxicity associated with RT for pituitary adenomas, with varying incidence across studies. In our cohort, hypopituitarism occurred in 40% of patients, which is higher than the rates reported in some studies but consistent with others that have longer follow-up periods. For example, Rieken et al. [24] reported hypopituitarism in 10.9% of patients, while Scheick et al. [25] found a rate of 26%. The higher incidence in our study may be due to the longer follow-up duration and more comprehensive endocrine monitoring, as late-onset hormonal deficiencies may develop years after treatment. This underscores the importance of long-term endocrine evaluation in patients undergoing RT for pituitary adenomas.

Visual impairment is a key concern in RT for pituitary adenomas due to the proximity of these tumors to critical visual structures, such as the optic chiasm and optic nerves. Across the reviewed studies, the incidence of visual toxicity varied but was consistently low, highlighting the advancements in RT techniques. In our study, the incidence of visual toxicity was exceptionally low at < 3%, the lowest among all reviewed studies. This outcome likely reflects the relatively moderate median dose administered (50.4 Gy), careful treatment planning, and the use of advanced techniques such as proton therapy, which offers superior dose conformity and precise energy deposition at the Bragg peak.Proton therapy’s ability to minimize radiation exposure to surrounding critical structures plays a pivotal role in reducing the risk of visual impairment, particularly in cases with tumors abutting or compressing the optic apparatus. Moreover, our cohort’s meticulous treatment planning and advanced imaging likely contributed to this outcome.

Radiation necrosis, brain-blood barrier disruptions and secondary malignancies were not observed in any of the studies, including ours. Rieken et al. [24] explicitly highlighted the absence of these complications, aligning with the results of Scheick et al. [25] and Sathe et al. [29]. These findings reflect the improved dosimetric precision and safety of fractionated RT techniques, particularly with modern technologies. Our study adds to this evidence, reinforcing that advanced RT approaches, including proton therapy, maintain a favorable safety profile even with long-term follow-up.

Proton therapy represents a significant advancement in RT, offering superior precision due to its unique dose deposition properties. Clinical studies have demonstrated that proton therapy achieves excellent tumor control rates while minimizing long-term complications. Wattson et al. [30] analyzed 165 patients with functional pituitary adenomas (FPAs) treated with proton therapy at a single institution over 20 years. The majority (92%) underwent proton stereotactic radiosurgery (PSRS), while the rest received fractionated proton therapy. Tumor control was excellent, with 98% of patients achieving local control at a median imaging follow-up of 43 months. Biochemical complete response varied by tumor subtype, with ACTH-secreting adenomas (Cushing’s disease and Nelson syndrome) achieving the best outcomes. In contrast, growth hormone- and prolactin-secreting adenomas demonstrated slower and less complete responses. Hypopituitarism was the most common toxicity, with 45% of patients developing new deficits at 3 years and 62% at 5 years. Larger target volumes were significantly associated with a higher risk of hypopituitarism. Other adverse effects included rare cases of seizures and cranial nerve palsies. The study concluded that proton therapy is an effective treatment for FPAs, particularly in cases resistant to surgery, offering strong tumor control with manageable late toxicities. Our results similarly emphasized the advantages of proton therapy in reducing toxicities and improving outcomes, particularly visual preservation and long-term tumor control.

## Conclusion

Proton therapy and other modern radiotherapy techniques have shown significant advantages in the treatment of pituitary adenomas by achieving durable tumor control rates with minimal long-term toxicities. Although only one-third of the patients received proton therapy, it demonstrated promising results in our cohort and should be validated in future studies.

This study highlights the role of accurate dose delivery, well-timed intervention and structured long-term follow-up in improving patient management and quality of life. Continued advancements in RT technology and treatment selection are expected to further reduce adverse effects while maintaining efficacy. Future research should focus on optimizing RT protocols, particularly for secretory tumors, and further exploring the long-term benefits of proton therapy in reducing toxicity and improving outcomes.

## Data Availability

No datasets were generated or analysed during the current study.

## Abbreviations

ACTH: Adrenocorticotrope hormone
CT: Computed tomography
CTCAE: Common Terminology Criteria for Adverse Events
FPA: Functional pituitary adenomas
FSH: Follicle-stimulating hormone
FSRT: Fractionated stereotactic radiotherapy
GH: Growth hormone
Gy: Gray
IMPT: Intensity-Modulated Proton Therapy
LC: Local control
LPFS: Local progression-free surival
MRI: Magnetic resonance imaging
OS: Overall survival
PFS: Progression-free survival
PSRS: Proton stereotactic radiosurgery
QoL: Quality of life
RT: Radiotherapy
SBO: Single-Beam Optimisation
SRS: Stereotactic radiosurgery
TSH: Thyroid stimulating hormone
3D-CRT: three-dimensional conformal radiation therapy
